# Identification and annotation of bovine granzyme genes reveals a novel granzyme encoded within the trypsin-like locus

**DOI:** 10.1007/s00251-018-1062-6

**Published:** 2018-06-08

**Authors:** Jie Yang, Christina Vrettou, Tim Connelley, W. Ivan Morrison

**Affiliations:** 1 The Roslin Institute, Royal (Dick) School of Veterinary Studies, University of Edinburgh, Edinburgh, EH8 9YL UK; 2 Institute of Immunity and Transplantation, Division of Infection and Immunity, University College London, Royal Free Hospital, London, NW3 2QG UK

**Keywords:** Granzyme, Cattle, Lymphocyte subsets, Genome loci, Enzymatic specificity

## Abstract

Granzymes are a family of serine proteases found in the lytic granules of cytotoxic T lymphocytes and natural killer (NK) cells, which are involved in killing of susceptible target cells. Most information on granzymes and their enzymatic specificities derive from studies in humans and mice. Although granzymes shared by both species show a high level of conservation, the complement of granzyme genes differs between the species. The aim of this study was to identify granzyme genes expressed in cattle, determine their genomic locations and analyse their sequences to predict likely functional specificities. Orthologues of the five granzyme genes found in humans (A, B, H, K and M) were identified, as well a novel gene designated granzyme O, most closely related to granzyme A. An orthologue of granzyme O was found in pigs and a non-function version was detected in the human genome. Use of specific PCRs demonstrated that all of these genes, including granzyme O, are expressed in activated subsets of bovine lymphocytes, with particularly high levels in CD8 T cells. Consistent with findings in humans and mice, the granzyme-encoding genes were located on three distinct genomic loci, which correspond to different proteolytic enzymatic activities, namely trypsin-like, chymotrypsin-like and metase-like. Analysis of amino acid sequences indicated that the granzyme proteins have broadly similar enzymatic specificities to their human and murine counterparts but indicated that granzyme B has a different secondary specificity. These findings provide the basis for further work to examine their role in the cytotoxic activity of bovine CD8 T cells.

**Electronic supplementary material:**

The online version of this article (10.1007/s00251-018-1062-6) contains supplementary material, which is available to authorized users.

## Introduction

The ability of CD8 T cells and natural killer (NK) cells to kill infected or abnormal target cells is an important functional characteristic of these cell types. Killing by CD8 T cells occurs upon recognition of antigenic peptides associated with class I MHC proteins, whereas NK cells utilise surface receptors that are activated as a consequence of altered expression of NK receptor ligands on the target cell surface. Although NK cells and CD8 T cells represent components of the innate and adaptive responses respectively, they utilise similar mechanisms to kill their target cells. Cell killing is achieved by release of the enzymatic contents of modified lysosomes known as lytic granules; release of perforin creates pores in the target cell membrane facilitating entry into the cell of granzymes, which act on various cellular pathways to trigger programmed cell death (Jenne and Tschopp 1988).

Granzymes are a family of serine proteases that exhibit various primary substrate specificities. Five granzymes—A, B, K, H and M—have been identified in humans. Mice express four of these granzymes—A, B, K and M—and six additional granzymes, C, E, D, F, G and N, which are not present in humans (Grossman et al. 2003). Granzymes have been classified into three distinct evolutionary groups, based on their primary substrate specificities, namely trypsin-like (granzymes A and K), chymotrypsin-like (granzymes B, H, C, E, M, D, F, G and N) and metase-like (granzyme M) (Smyth et al. 1996). The classification into these three groups also corresponds to the distribution of the genes in three different chromosomal locations (Trapani 2001). Mapping of these genomic regions has revealed that other related serine proteases such as the cathepsin G and mast cell proteases are closely linked with granzymes B and H in the chymotrypsin locus, while the metase locus holds a cluster of neutrophil elastase genes, including azurocidin 1 (ZAU1), proteinase 3 (PRTN3), neutrophil expressed (ELANE) and complement factor D (CFD), 185 to 310 downstream of granzyme M (Pilat et al. 1994; Grossman et al. 2003)

Consistent with granzymes being structurally related to chymotrypsin, studies of human and mouse granzymes have defined a number of common features, including a shared consensus sequence at the N-terminus (Bleackley et al. 1988; Jenne and Tschopp 1988; Murphy et al. 1988), the presence of short pro-peptides that are cleaved to produce the enzymically active protein, three conserved amino acids that determine catalytic activity (the catalytic triad—His-57, Asp-103 and Ser-195) (Murphy et al. 1988) and three to four disulfide bridges (Smyth et al. 1996; Trapani 2001). The substrate specificity of the different granzymes is dependent on the three-dimensional structure of the substrate-binding pocket (Perona and Craik 1995), which is determined by variation in key amino acid residues in and around the pocket (Smyth et al. 1996).

Current knowledge of the complement of granzyme genes and their biological activities is based largely on information from humans and mice. Studies of the role of granzymes in cell killing have focused largely on granzymes A and B and, in the case of granzyme B, have revealed differences between mice and humans in the pathways by which this granzyme mediates apoptosis of target cells. In cattle, CD8 T cells exhibiting cytotoxic activity have been shown to be key mediators of immunity to the intracellular protozoan parasite *Theileria parva*. As a first step in studying the mechanism of killing of parasitized cells, we set out to identify and annotate the granzyme genes expressed in cattle. The results demonstrate that the complement of granzyme genes and their genomic organisation are similar to that of humans but that cattle express an additional novel trypsin-like granzyme, which we have designated granzyme O.

## Materials and methods

### Bovine granzyme genome analysis

Nucleotide sequences of bovine granzymes were identified in the bovine genome assembly, UMD3.1, using the nucleotide-nucleotide basic local alignment search tool (BLASTN) with cDNA sequences of human and mouse granzyme genes obtained from National Center for Biotechnology Information (NCBI) RefSeq database: Human: GzmA (NM_006144), GzmB (NM_004131), GzmH (NM_033423), GzmK (NM_002104) and GzmM (NM_005317); Mouse: GzmA (NM_010370), GzmB (NM_013542), GzmK (NM_008196), GzmC (NM_010371), GzmE (NM_010373), GzmD (NM_010372), GzmF (NM_010374), GzmG (NM_010375), GzmN (NM_153052) and GzmM (NM_008504). Use of the obtained genome sequences to search the bovine expressed sequence tags (EST) database identified the corresponding cDNA sequences.

### Sequence analysis

Sequence analyses such as ClustalW alignment and amino acid translations were performed using the DNAsis Max V2.7 programme (MiraiBio, Alameda, CA, USA). Prediction of the signal sequence cleavage site was performed using an algorithm previously described (von Heijne 1986). Residues involved in the catalytic triad and disulfide bridge formation were analysed by EBI PPsearch (http://www.ebi.ac.uk/Tools/ppsearch/) and Prosite (http://www.expasy.org/prosite/).

### Chromosomal location analysis

Trypsin-like, chymotrypsin-like and metase loci were identified and mapped using the following human and mouse nucleotide sequences from the NCBI database (in addition to the granzyme sequences listed above): Human: CMA1 (NM_001836), CTSG (NM_001911), AZU1 (NM_001700), PRTN3 (NM_002777), ELANE (NM_001972) and CFD (NM_001928); Mouse: Cma1 (NM_010789), Mcpt1 (NM_008570), Mcpt9 (NM_010782), Mcpt2 (NM_008571), Mcpt4 (NM_010779), Mcpt8 (NM_008572), Ctsg (NM_007800), Prtn3 (NM_011178), Elane (NM_015779) and Cfd (NM_013459). Bovine orthologues were identified using BLASTN searches in the bovine genome assembly, UMD3.1 (in addition to the granzyme sequences identified): Cattle: CMA1a (corrected (corr)_ENSBTAG00000027033), CMA1b (corr_ENSBTAG00000037578), CTSG1 (corr_ENSBTAG00000013234), CTSG2 (ENSBTAG00000040134), DDN1 (NM_174296.2), DDN2 (corr_ENSBTAG00000038159), DDN3 (corr_ENSBTAG00000013055), DDN4 (corr_ENSBTAG00000038080), ZAU1 (ENSBTAG00000045829), PRTN3 (corr_ENSBTAG00000046105), ELANE (ENSBTAG00000046188) and CFD (ENSBTAG00000048122). The chromosomal locations of the orthologues in the bovine genome were annotated by gene mapping using the identified sequences.

### Phylogenetic analysis

Phylogenetic analysis was performed on the nucleotide sequence of predicted bovine functional granzyme genes and their related human, mouse and pig genes (accession numbers for the sequences used are described above). The relationships across species were established by analysing a ClustalW sequence alignment with the Neighbour-joining method using MEGA7.0 software (Kumar et al. 2016). Percentage bootstrap values were obtained from the mean of 2000 replications. The evolutionary distances were computed using the Maximum Composite Likelihood method and are in the units of the number of base substitutions per site. All positions containing gaps and missing data were excluded.

### Amplification of granzyme and perforin transcripts from cDNA by RT-PCR

Total RNA was extracted from freshly harvested bovine peripheral blood mononuclear cells (PBMC) and from CD4, CD8 and γδ T cells purified from *T. parva*-specific T cell lines (Goddeeris and Morrison 1988) derived from cattle that had been immunised against *T. parva* by infection and treatment as described previously (Radley et al. 1975) and shown to generate strong MHC-restricted cytotoxic T cell responses. Activated NK cells were purified from PBMC of *T. parva*-naïve cattle by cell sorting, using an NKp46-specific monoclonal antibody, and stimulated in vitro with irradiated *T. parva*-infected cells in the presence of recombinant human IL-2 as described previously (Connelley et al. 2014). The resultant cells were again purified to > 98% purity by cell sorting prior to RNA extraction. The T and NK cells were harvested 5–7 days after the most recent antigenic stimulation in vitro. RNA was extracted using Tri-reagent (Sigma-Aldrich, UK) and cDNA synthesised using the Reverse Transcription System (Promega, USA) with priming by the Oligo (dT)15 primer; both kits were used according to the manufacturer’s instructions. PCR assays were developed to amplify full-length coding regions for bovine granzymes and perforin based on sequences identified from bovine genomic and EST databases. PCR primers were designed either manually or using the Primer3 programme and synthesised by MWG biotech (Ebersberg, Germany). Primer sequences are shown in Table 1. PCR reactions were composed of 10 pmol of primers, 0.5 units BIOTAQ (Bioline, UK), 2 μl SM-0005 buffer (ABgene, UK), 1 μl cDNA (0.05 μg/μl) in DDW and nuclease-free water to give a final volume of 20 μl. The programme used was as follows: 94 °C for 3 min, 30 cycles (94 °C for 1.5 min, 55 °C for 1.5 min, 72 °C for 1.5 min) and a final extension period of 72 °C for 10 min. The PCR products were sub-cloned into the pGEM-T vector and nucleotide sequencing performed by DBS Genomic (Durham University).

**Table 1:** PCR primers designed for detection of bovine granzymes and perforin

Primers	Sequences (5′–3′)
Granzyme A (For)	ATTGATTGATGTGGGGACAC
Granzyme A (Rev)	AAAAAGTAACAGCAAATGAAATACAA
Granzyme O (For)	AGTCTCCATATGTGAATAACAGGAG
Granzyme O (Rev)	CCCTTTCACTTGGTTACTTCG
Granzyme B (For)	CATCCTGGGCAGTCTTTCTA
Granzyme B (Rev)	CCTGCAGTGTGATTCTGGAT
Granzyme H (For)	CTGACCTGGGCAAATCTTCT
Granzyme H (Rev)	GGACAATGGTCAGTGCAGAG
Granzyme K (For)	TTCCTTTGCCAATACAGTCAG
Granzyme K (Rev)	AGCAGCTGATAGAGCCAAGA
Granzyme M (For)	GAGGCCCCCCAGATCCAAG
Granzyme M (Rev)	CCCCTTGGAACACAGAATCA
Perforin (For)	CAGGGTGGTCAAGCTAGAGG
Perforin (Rev)	AGGTGAGGCAAGCATTTGAC

## Results

### Identification of bovine granzyme genes

BLASTN searches of the bovine genome assembly, UMD3.1, identified six putative granzyme genes predicted to encode full-length functional proteins. These included genes orthologous to the five granzyme genes found in humans (A, B, K, H and M) and a further gene with no close orthologue in any species but most closely related to granzyme A (Table 2). We have designated this novel nucleotide sequence (genome reference ENSBTAG00000027865) Granzyme O (following previous alphabetical allocation of letter symbols). Sequences matching each of the bovine granzyme genes identified from the genome were found within the GenBank bovine EST database: granzyme A (EH165320.1), granzyme O (CN787157.1), granzyme B (DN533698.1), granzyme H (CK776010.1), granzyme K (FE012584.1) and granzyme M (DV805053.1). Searches of this database using other murine granzyme gene sequences did not reveal any additional bovine granzymes. Consistent with findings in other species (Sattar et al. 2003), comparative analysis of the sequences showed that each of the bovine granzymes was more closely related to their counterparts in human and mouse than other bovine granzyme genes, with levels of 76–83% nucleotide similarity and 70–77% amino acid identity with the respective human genes and 70–75% nucleotide similarity and 64–71% amino acid identity with the respective mouse granzymes.

**Table 2:** Summary of functional granzyme genes identified in different species

Species	Granzyme											
	A	O	B	H	K	C	E	D	F	G	N	M
Human	+	–	+	+	+	–	–	–	–	–	–	+
Murine	+	–	+	–	+	+	+	+	+	+	+	+
Cattle	+	+	+	+	+	–	–	–	–	–	–	+

Cattle genome reference (UMD3.1): granzyme A (ENSBTAG00000021958), granzyme O (ENSBTAG00000027865), granzyme B (ENSBTAG00000010057), granzyme H (ENSBTAG00000010828), granzyme K (ENSBTAG00000005164) and granzyme M (ENSBTAG00000002100)

### Validating expression of bovine granzymes and perforin by RT-PCR analyses

**Fig. 1:**
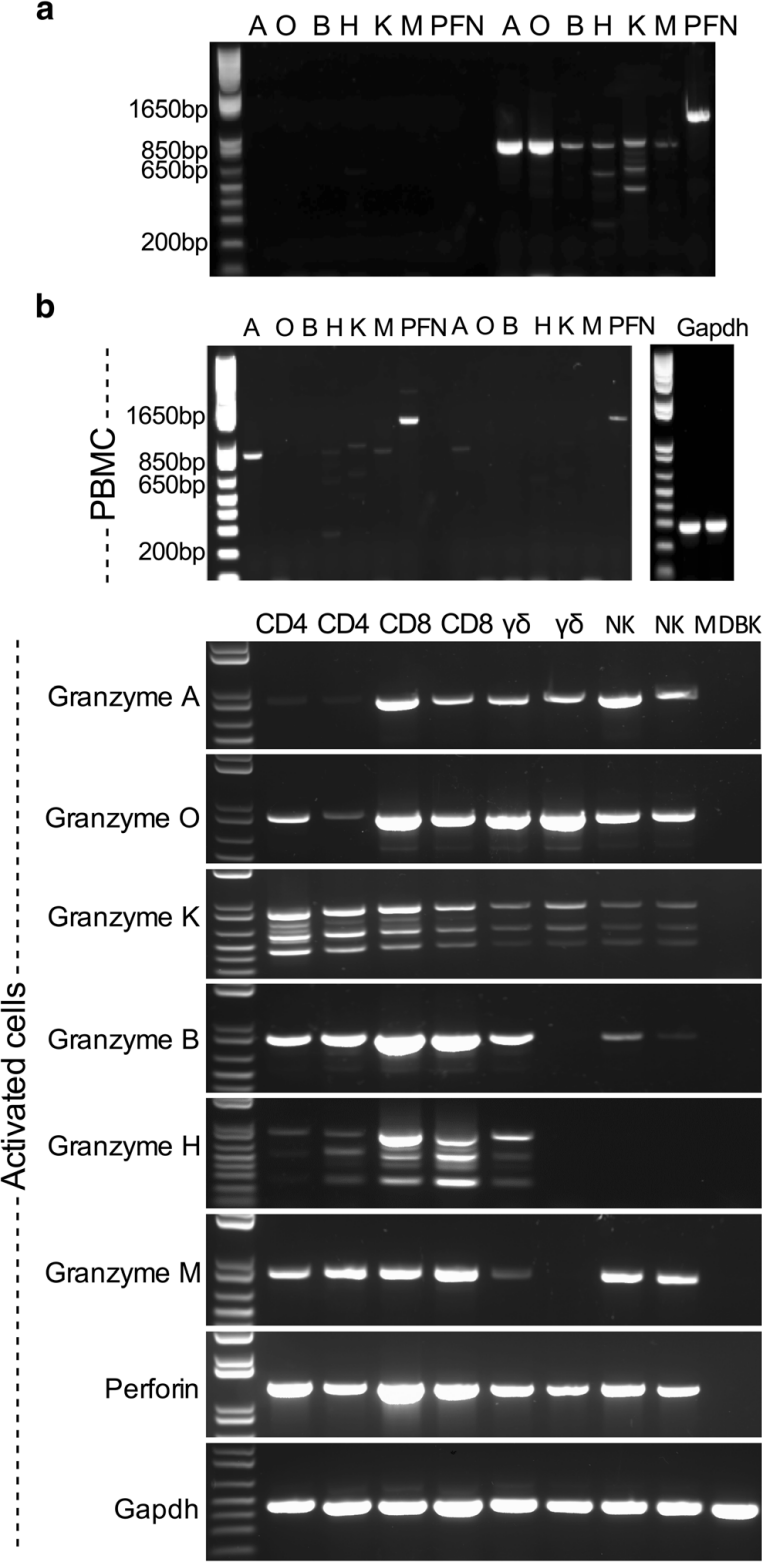
PCR products obtained using primers designed to be specific for each of the bovine granzyme genes and perforin (PFN). The sizes of the PCR products: granzyme A (A), 838 bp; granzyme O (O), 849 bp; granzyme B (B), 818 bp; granzyme H (H), 820 bp; granzyme K (K), 889 bp; granzyme M (M), 833 bp; PFN, 1275 bp. **a** A *T. parva*-specific CD8 T cell line (purity, 99%) harvested 7 days after antigenic stimulation was used as the source of template cDNA. Lanes on the left contain primers without template for the individual granzymes and those on the right include template cDNA. **b** Resting bovine peripheral blood mononuclear cells (PBMC) and purified populations (purity, > 98%) of activated T cell subsets (CD4, CD8 and γδ T cells) and activated NK cells harvested 7 days after antigenic stimulation were used as the source of template cDNA. As a control, cDNA isolated from Madin-Darby Bovine Kidney (MDBK) epithelial cells was included

Based on the sequences identified above, pairs of PCR primers were designed to amplify the full-length coding region of each of the granzymes, as well as the perforin gene, which had been identified previously (genome reference XM_585583). PCR assays with these primers detected transcripts of the expected sizes for all six granzyme genes and perforin in cDNA prepared from bovine cytotoxic CD8 T cell lines (Fig. 1a). A single band was obtained for all except granzymes H and K, which each gave three bands, one of the predicted size and two smaller bands. Sequencing of subclones prepared from the PCR products confirmed that they were all identical to those originally identified from the bovine genome. The additional bands obtained for granzymes H and K were found to represent alternatively spliced forms. The two smaller products of granzyme H were missing exon 4 (562 bp) and exons 2, 3 and 4 (265 bp), respectively, and those of granzyme K were missing exon 4 (619 bp) and exons 3 and 4 (468 bp), respectively. In conclusion, all of the identified bovine granzyme genes are expressed at the mRNA level in activated bovine CD8 T cells.

### Granzyme expression by subsets of activated lymphocytes

In humans and mice, granzymes are expressed not only by CD8 T cells but also other T cell subsets and NK cells (Anthony et al. 2010; Bovenschen and Kummer 2010). They are expressed at variable levels in resting cells and are upregulated upon activation. Resting bovine PBMC and purified populations of activated T cell subsets (CD4, CD8 and γδ T cells) and activated NK cells were examined by PCR for expression of the different granzymes. The results are shown in Fig. 1b. Transcripts of granzymes A, K, H and M were detected in resting PBMC, but granzymes O and B were not detectable. The activated CD8 T cells expressed transcripts for all six granzymes, B, A and O being particularly abundant. Similarly CD4 T cells expressed all granzymes, but the bands obtained for granzymes H, A and O were much weaker than those obtained for granzyme B. Divergent results were obtained for the two γδ T cell lines; while both lines expressed similar levels of the trypsin-like granzymes (A, O and K), A and O being particularly abundant, one of the lines yielded no or weak bands for B, H and M, whereas clear bands were detected for the other line. The NK cell lines gave no (granzyme H) or weak (granzyme B) bands for the chymotrypsin-like granzymes but yielded clear bands for the remaining granzymes. The alternatively sliced forms of granzymes K and H were detected in all lymphocyte subsets where transcripts were present.

### Chromosomal location of granzyme genes

Annotation of the bovine genome using the identified bovine granzyme orthologues revealed that, similar to human and mouse, the bovine granzyme genes are found in three loci on three different chromosomes:

#### Trypsin-like locus

The trypsin-like locus located on chromosome 20 contains the functional granzyme genes A and K, which are separated by 74 kb on chromosome 20. The orientation and arrangement of the genes are similar to that in humans and mice. The bovine locus contains an additional granzyme gene, located between the A and K genes, designated granzyme O. To date, there are no reports of a close orthologue of granzyme O in any other species. However, a BLASTN search of the pig genome (Sscrofa11.1) revealed a full-length open reading frame (ENSSSCG00000016902) that shows 91% similarity in the coding region to the cattle gene. The EST sequence data in both species (pig EST database reference BW979091.1), together with RT-PCR analysis of cattle T cells in this study, indicate that granzyme O is expressed. Analysis of genome DNA sequence in the region between the granzyme A and K genes in human and mouse revealed an unprocessed pseudogene in human (ENSG00000249454.1) and a processed pseudogene in mouse (ENSMUSG00000051002), which exhibit 89 and 66% nucleotide similarity to cattle granzyme O gene, respectively (Supplementary 1B). The human granzyme O-like pseudogene comprises four exons that contain several premature stop codons, whereas the mouse granzyme O-like pseudogene contains only 259 bp of DNA sequence, which based on alignments appears to correspond to exon 4 in the bovine gene (Supplementary 1A). Phylogenetic analysis of the nucleotide sequences of bovine, human, pig and murine granzyme A, granzyme K and granzyme O identified three distinct subgroups segregated according to granzyme gene, with the O subgroup being more closely related to A than K (Supplementary 1C). Interspecies comparisons showed that the bovine genes are more closely related to the pig orthologues. The results suggest that the O gene diverged prior to speciation, possibly as a result of duplication of granzyme A, and that it subsequently became non-functional in human and mouse.

#### Chymotrypsin-like locus

A preliminary map of the cattle chymotrypsin-like locus, based on a previous version of the bovine genome assembly (NCBI, Build 2.1 database released on October 2005), has been reported (Gallwitz et al. 2006). However, the updated cattle genome contains changes in the assembly of this region; hence, the locus has been re-drawn according to the version of Ensembl, UMD3.1 (released on Nov 2009). The locus is on chromosome 21 in cattle. Although its overall size (156 kb) is similar to that in human (129 kb), the number of annotated functional genes in cattle (10) is more than twice that found in human.

**Fig. 2:**
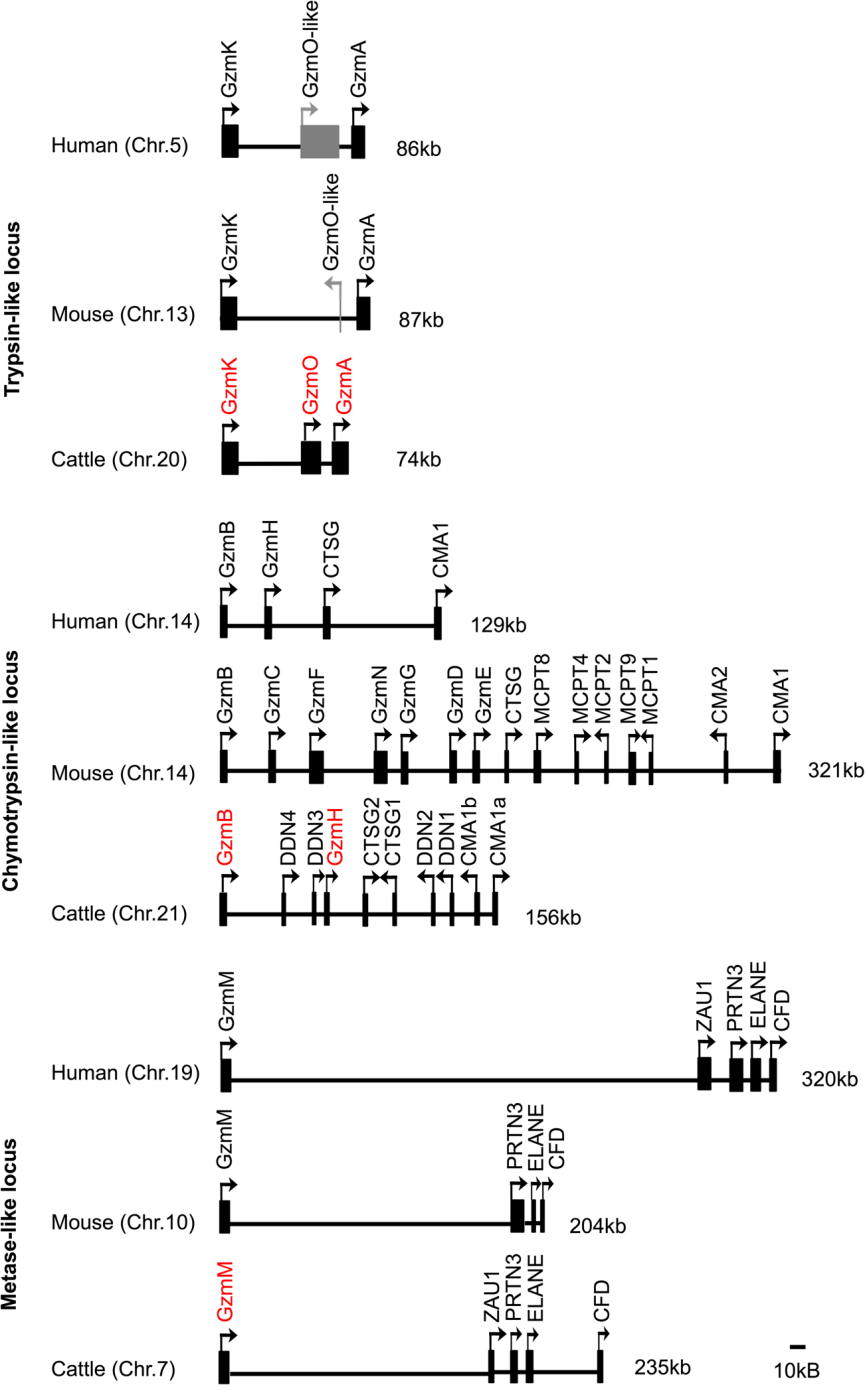
Comparison of the genomic organisation of the human, murine and cattle granzyme loci. Human and murine loci were drawn based on the genome assembly of GRCh38.p12 and GRCm38.p6, respectively. Three corresponding loci of trypsin-like (chr 20: 24,033,747–24,107,687), chymotrypsin-like (chr 21: 35,112,322–35,267,979) and metase-like (chr 7: 44,797,942–45,032,845) were drawn based on bovine genome assembly (UMD3.1 (GCA_000003055.3). Bars indicate gene positions; arrows indicate transcriptional orientation; numbers on the right-hand side of loci indicate the length of locus. Intervals between genes are drawn to scale. Bovine granzyme genes are highlighted in red. Functional genes are shown in black boxes and non-functional genes in grey. Chr = chromosome; Gzm = granzyme; CTSG = cathepsin G; CMA = mast cell a-chymase; DDN = duodenase; ZAU1 = azurocidin 1; PRTN3 = proteinase 3; ELANE = neutrophil elastase preproprotein; CFD = complement factor D

**Fig. 3:**
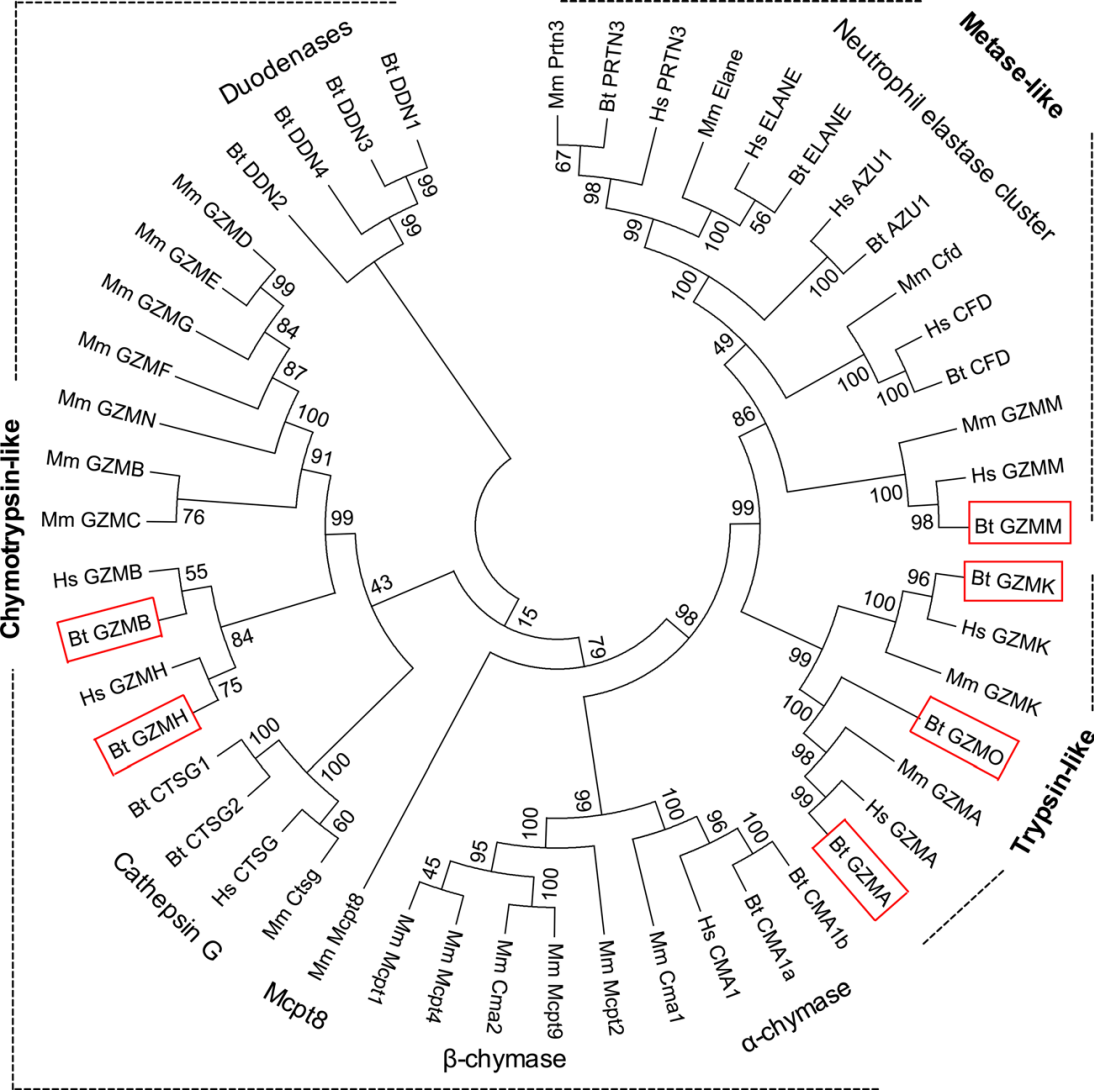
Phylogenetic relationship of granzymes and their related genes from human, mouse and cattle. Phylogenetic analysis was performed on cDNA sequences and the tree was constructed with the neighbour-joining algorithm using Mega 7.0 software. Numbers represent percentage bootstrap values out of 2000 replications. The evolutionary distances were computed using the Maximum Composite Likelihood method and are in the units of the number of base substitutions per site. All positions containing gaps and missing data were eliminated. The three main phylogenetic groups are labelled as trypsin-like, chymotrypsin-like and metase. Bovine granzyme genes are highlighted in red boxes. Hs, *Homo sapiens*; Mm, *Mus musculus*; Bt, *Bos Taurus*; GZM, granzyme; CTSG, cathepsin G; CMA, mast cell a-chymase; DDN, duodenase; ZAU1, azurocidin 1; PRTN3, proteinase 3; ELANE, neutrophil elastase preproprotein; CFD, complement factor D

The cattle chymotrypsin-like locus contains two α-chymase genes (Cma1a and Cma1b) and two cathepsin G genes (Ctsg1 and Ctsg1) (Fig. 3), compared to only one gene of each in human and mouse. Each pair of genes shows a high level of predicted amino acid identity (94 and 78%, for Cma and Ctsg, respectively). A further four related genes sharing a high level of nucleotide similarity with granzyme B (69.8–72%) were found, two lying between the granzyme B and granzyme H genes and the other two lying between the Ctsg and Cma1 genes. However, none of them appear to be paralogous to granzyme B. One of these genes (DDN1) was identified previously as a duodenase (Zamolodchikova et al. 1995). The three additional genes show close sequence similarity to DDN1 and to each other (Fig. 3) and therefore are considered to be members of the duodenase family, designated DDN2–DDN4 according to their order in the genome (Fig. 2). So far, duodenase genes have only been reported in ruminants (McAleese et al. 1998).

#### Metase locus

The bovine granzyme M gene is located on chromosome 7 and, as in humans and mice, is linked to a neutrophil elastase gene cluster 165 kb downstream of this gene.

### Phylogenetic relationships between granzymes and related enzymes

A phylogenetic tree constructed from the nucleotide sequence of all functional granzymes and their related genes from human, mouse and cattle is shown in Fig. 3 and the levels of amino acid identity between the granzymes are presented in Table 3. In general, these data demonstrate divergence into three broad groups, corresponding to the trypsin-like, chymotrypsin-like and metase proteins and to the different chromosomal locations of the genes. This is reflected by much lower sequence identity between the groups (up to 44%) than within groups (45–91%).

**Table 3:**
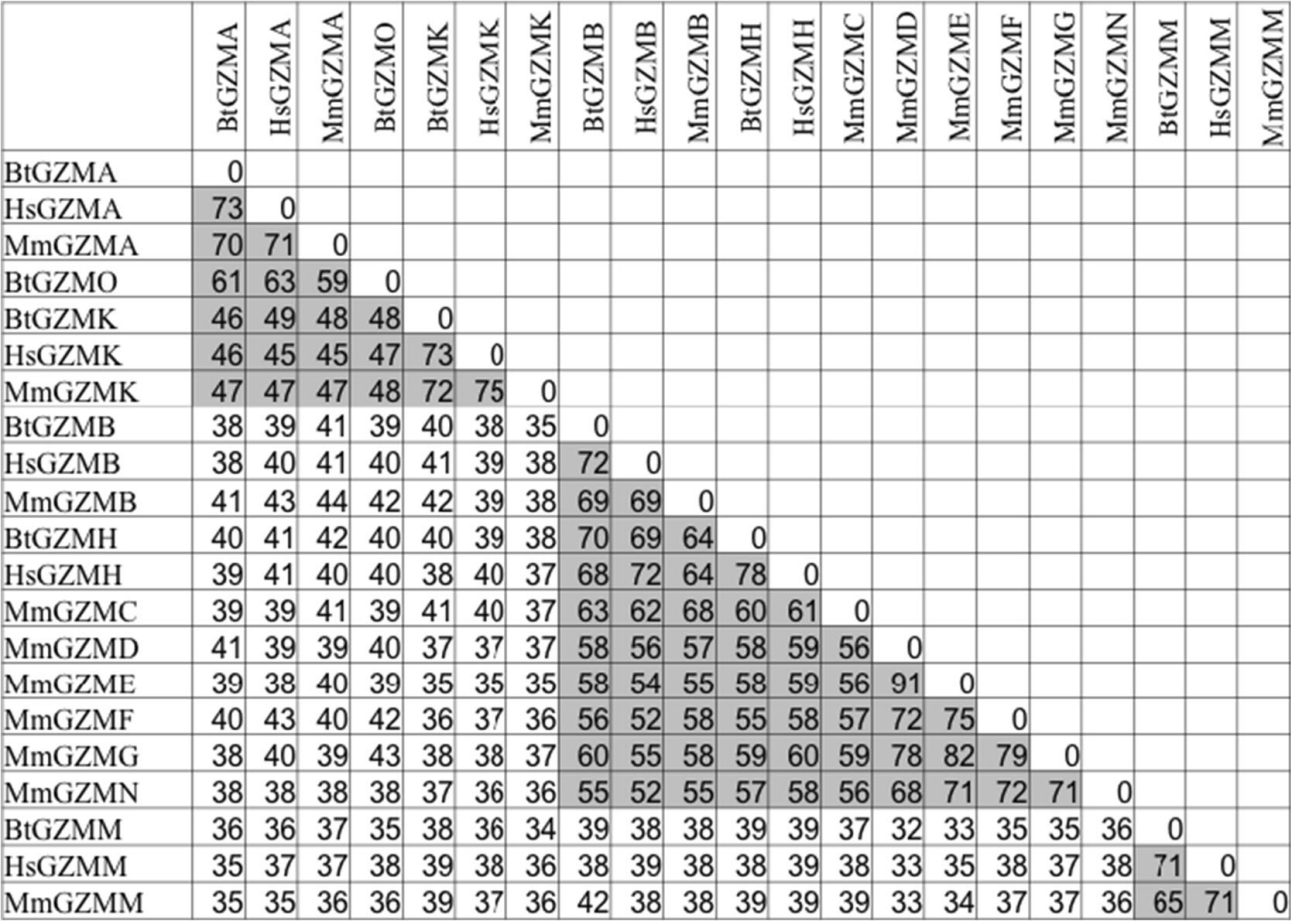
Percentage amino acid identities between mature protein sequences of bovine, human and murine granzymes

ᅟ

Comparisons are based ClustalW alignment with manual adjustments for deletions and insertions. Numbers representing the amino acid identity of granzymes within each subgroup are highlighted in grey

*Hs Homo sapiens*, *Mm Mus musculus*, *Bt Bos Taurus*, *GZM* granzyme

### Characteristic structural features of granzyme proteins

**Fig. 4:**
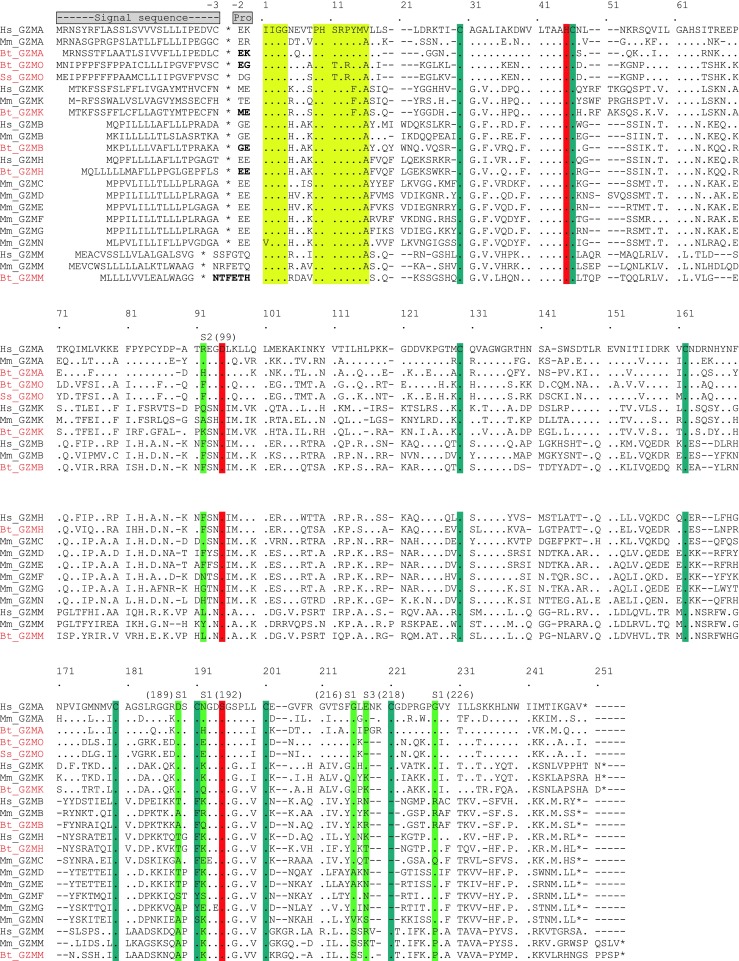
Amino acid sequence alignment of bovine, human, mouse and pig granzymes showing conservation of functionally relevant amino acid residues. Signal sequence cleavage sites are indicated by an asterisk; conserved consensus sequences at the N-terminus at positions 1–4 (IIGG) and 9–16 (PHSRPYMA) are shown in yellow; the three amino acids of the catalytic triad are shown in red; cysteine residues that form disulfide bonds are shown in blue. Substrate-determining residues in S1, S2 and S3 are shown in green. Numbering of residues is based on that used for the chymotrypsinogen amino acid sequence. Dot, identical; dash, gap. Hs, *Homo sapiens*; Mm, *Mus musculus*; Bt, *Bos Taurus*; Ss, *Sus scrofa*; GZM, granzyme

Multiple alignments of the predicted amino acid sequences of human, mouse and cattle granzymes were analysed to investigate their structural homologies and predicted substrate-binding specificities (Fig. 4). Sequence analysis revealed features of cattle granzymes typical of the murine and human orthologues: namely, highly conserved consensus sequences at positions 1–4 (IIGG) and 9–16 (PHSRPYMA); conserved signal sequence cleavage sites prior to the propeptide (von Heijne 1986; Pilat et al. 1994; Smyth et al. 1995; Kelly et al. 1996; Sayers et al. 1996); and conserved amino acid residues His-45, Asp-95 and Ser-195, which form a catalytic triad, and sites for three to four disulfide bonds, Cys30-Cys46, Cys162-Cys180, Cys191-Cys221 and Cys130-Cys201.

Previously obtained data on the structure of murine and human granzymes (Perona and Craik 1995) and the use of homology molecular models provide the opportunity to predict the substrate specificity of granzymes from other species by analysis of the corresponding specificity-determining residues. Residues 189, 192, 216 and 226 (based on chymotrypsinogen numbering), important for primary substrate specificity (Odake et al. 1991; Kam et al. 2000), deduced in the cattle granzymes as well as pig granzyme O in this model, are shown in Table 4.

**Table 4:**
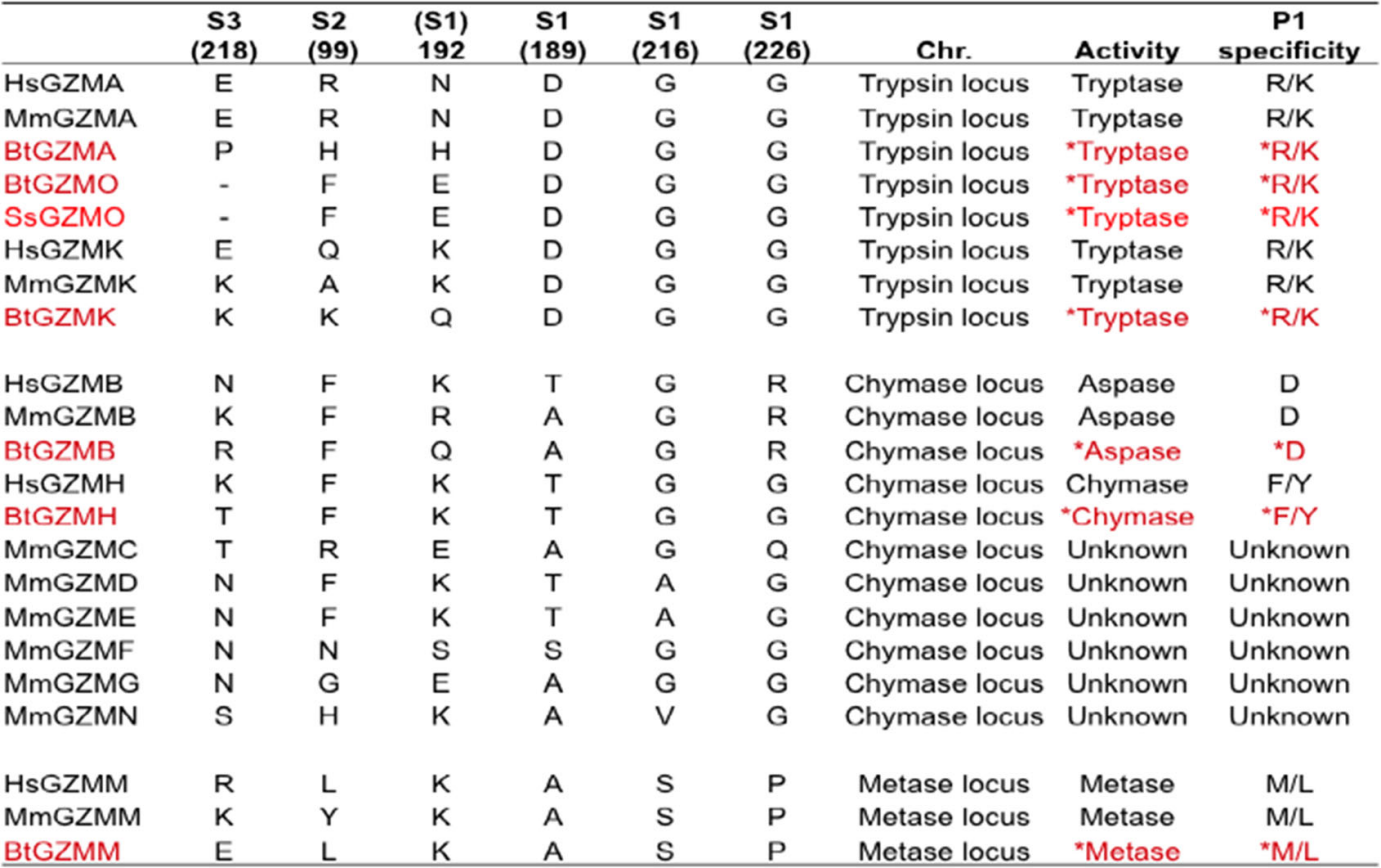
Comparison of key amino acid residues in the substrate-binding site and predicted enzymatic specificities of granzymes from different species

ᅟ

Based on data from (Odake et al. 1991; Kam et al. 2000; Sattar et al. 2003), with additional data on bovine and pig in red from the present study. P1 (the residue of the substrate) specificity refers to primary substrate specificity, which is determined by primary substrate-binding pocket called S1 binding sites of the protease. Extended substrate specificity is determined by secondary substrate-binding pockets, called S2 and S3 binding sites of the protease. The predicted enzymatic functions and P1 specificity of cattle and pig granzymes are highlighted by asterisk. The sequence residue numbering is based on the chymotrypsinogen numbering

*Hs Homo sapiens*, *Mm Mus musculus*, *Bt Bos Taurus*, *Ss Sus scrofa, GZM* granzyme

The presence of a negatively charged residue Asp189 is of great importance in determining the primary substrate specificity to trypsin-like enzymes (Ruhlmann et al. 1973). This amino acid is positioned at the bottom of S1 pocket and electrostatically interacts with the basic residue at P1 in the substrate. Gly residues at positions 216 and 226 help to provide access of the bulky lysine or arginine in the substrate to Asp189. As cattle granzyme A, O and K and pig granzyme O share the identical specificity-determining triplet (Asp189-Gly216-Gly226) with their human and mouse counterparts, these novel enzymes most likely have the trypsin-like specificity and prefer to cleave after Arg or Lys residues.

In chymotrypsin-like enzymes, the crucial role is shifted to the amino acid on position 226 that is situated at the back of the specificity pocket (Caputo et al. 1994; Edwards et al. 1999). This is associated with the absence of a disulfide bond in the vicinity of the enzyme active site, compared with other granzyme groups (Zamolodchikova et al. 2003). The small size of the uncharged residue Gly226, present in all chymotrypsins, is thought to permit bulky hydrophobic side chains to be accommodated and cleaved (Edwards et al. 1999). Granzyme H has no direct rodent counterpart but is reported to have the typical chymotrypsin-like activity to cleave after Phe or Tyr at P1. The presence of conserved amino acids at Thr189, Gly216 and Gly226 (S1 subsite) in cattle granzyme H is predictive of chymotrypsin-like activity. However, the existence of a positively charged residue, Arg226, in bovine granzyme B suggests a unique specificity for the negatively charged acidic side chain of Asp in the substrate (Poe et al. 1991; Caputo et al. 1994; Waugh et al. 2000). As the Asn189-Gly216-Arg226 amino acid triplet within the S1 pocket of cattle is highly conserved with those in human and mouse, this enzyme is predicted to act on substrates containing the acidic residue Asp in P1.

Structural studies of chymotrypsin A have indicated that the substrate specificity of granzyme M for the carboxyl terminal of long narrow hydrophobic amino acids, Met and Leu, is dependent on two key residues, Lys192 and Ser216 (Smyth et al. 1996). The presence of conserved amino acids Lys192 and Ser216 in cattle granzyme M is consistent with it having metase specificity similar to the human and murine counterparts.

## Discussion

The studies reported in this paper have identified six classes of granzymes in cattle, A, O, B, K, H and M, based on analysis of genome and EST databases. Specific PCR protocols have been developed and optimised for all of these granzymes. Analysis of cDNA from *T. parva-*specific CD8 T cell lines using these PCR assays showed that all of the enzymes are expressed at the RNA level in activated CD8 T cells. Consistent with findings in humans and mice, the granzyme-encoding genes were located on three different loci within the genome, which correspond to different proteolytic enzymatic activities, namely trypsin-like, chymotrypsin-like and metase. Analysis of primary amino acid sequences indicated that the granzyme proteins have enzymatic specificities similar to their human and murine counterparts.

The identified granzymes included a novel transcribed granzyme, termed granzyme O, not previously reported among the granzymes described in any species. An orthologue was also identified in the pig and non-functional genes or gene fragments were present in human and mouse, indicating that this novel gene evolved by gene duplication before the human-mouse-cattle-pig species divergence and subsequently became non-functional in humans and mice.

Activated NK and T lineage cells, including CD4, CD8 and γδ T cells, are known to be the dominant cellular source of all granzymes in human and mouse (Anthony et al.; Bovenschen and Kummer). Preliminary comparison of gene expression in cDNA prepared from purified activated lymphocyte sub-populations confirmed expression of all granzyme genes in both the CD4 and CD8 T cells, although the levels of expression of most granzymes were higher in the CD8 T cells. RNA expression of granzyme M was detected in all activated lymphocyte sub-populations, in particular CD4 and CD8 T cells, which is consistent with the finding by De Koning showing that human granzyme M is expressed by lymphocytes of both the innate and adaptive immune system (de Koning et al. 2010), although other studies in human reported granzyme M are strictly expressed in the γδ and NK cells (Sayers et al. 2001). The divergent pattern of expression observed for the two preparations of γδ T cells examined, one showing expression of all granzymes but the other showing little or no expression of chymotrypsin-like (B and H) and metase-like (M) granzymes, clearly indicates functional heterogeneity within this subset, which requires further investigation. The NK cells showed very low levels of expression of the chymotrypsin-like granzymes but clear expression of the other granzymes. In contrast to the study in human showing abundant expression of granzyme H in human NK cells (Sedelies et al. 2004), non-detectable RNA expression of granzyme H in bovine NK cells indicated species-dependent differential expression of this granzyme. In addition to the full-length transcripts, truncated, alternatively spliced versions of granzymes H and K were detected in all cell subsets. The shortest transcript of each is predicted to be non-functional, since the missing exons include codons for one or two of the amino acids that make up the catalytic triad. The functional activity of the other truncated trancripts that only lack exon 4 remains unclear.

Annotation of the genome loci containing the bovine granzyme genes has demonstrated that the gene organisation is similar to that in human and mouse. In each species, the genes are found in three distinct chromosomal locations, each of which has similar gene organisation across the species. Analysis of phylogenetic trees has revealed the homologous relationship between each subgroup of cattle genes and their human and mouse counterparts. Comparison of mature protein sequences of all granzymes from human, mouse and cattle has shown obviously higher levels of identity within each group between species (45–91%) than between the groups (up to 44%). All of these findings support the view that the granzyme family of genes in the different species have evolved from common ancestors and that differences in gene complement have arisen mainly due to differential deletion or loss of function. Thus, unlike cattle, humans and mice have lost granzyme O and in contrast to the other species mice have lost granzyme H. In place of the latter, mice have an additional set of genes encoding granzymes C, D, E, F, G and N, which although no remnants are found in other species may also have been lost in a common ancestor of these species by a gene deletion event.

The gene content and organisation of the trypsin-like and metase loci were similar to those in humans and mice. In contrast, the chymotrypsin-like locus has expanded in gene content due to duplication of non-granzyme genes (α-chymase and cathepsin G) and the presence of an additional set of closely related genes (duodenases), not detected either in the chymotrypsin locus or elsewhere in the human or mice genomes. These findings together with the expanded chymotrypsin-like locus of mice including the presence of Mcpt8 family and multiple β-chymases and granzymes in mouse (Gallwitz and Hellman 2006) further illustrate the extensive changes that have occurred in this locus following species divergence.

Analysis of the amino acid sequences of bovine granzymes in relation to data from homology molecular models of known crystal structures of human or murine orthologues enabled us to explore the likely substrate specificity of the bovine granzymes. These initial analyses indicate that they are likely to have primary specificities comparable with their human and mouse counterparts but with some differences in their extended substrate specificities.

To date, crystal structures of human granzyme A, B, K, H and M have been reported by several groups (Estebanez-Perpina et al. 2000; Hink-Schauer et al. 2002, 2003; Bell et al. 2003; Wu et al. 2009; Wang et al. 2012). The crystal structures, coupled with results of biological experiments and structural modelling, have provided information on residues that are critical for interacting with the substrates. For example, a structural study of granzyme A has shown that the specificity for a basic residue of the S1 pocket is primarily due to the side chain of Asp189 at the bottom of the pocket (Bell et al. 2003), whereas the primary specificity of granzyme B for Asp occurs through a salt bridge with Arg226, anchored at the back of the S1-specificity pocket of granzyme B (Estebanez-Perpina et al. 2000; Waugh et al. 2000). Crystal structure comparison of the active form of granzyme M with the inactive Asp86Asn-GzmM mutant has revealed that a peptide loop (L3 residues 214–226) is most important in determining substrate specificity (Wu et al. 2009). Mahrus and Craik (2005) provided experimental evidence to support the respective substrate specificities of human granzyme A, B, K, H and M, using positional scanning of synthetic combinatorial libraries of four-amino acid peptides. The results of these studies indicated that homology molecular modelling is a reliable and productive method for predicting differences in fine substrate specificity of related granzymes and novel orthologues of the same granzyme. Comparison of the substrate-determining amino acid residues in humans and mice with corresponding residues in bovine granzymes predicts that cattle granzymes have primary substrate specificities identical to their human and mouse counterparts. The findings indicate that the novel granzyme O is most likely to have trypsin-like activity, consistent with the specificity of the other granzymes (A and K) in the same gene locus. The trypsin-like specificity of granzyme A, O and K is also consistent with the idea that these genes arose by duplication prior to divergence of the respective mammalian species.

Although both human granzymes A and K have the trypsin-like specificity characterised by cleavage after basic residues Arg or Lys (Mahrus and Craik 2005), they display highly restricted substrate specificities that only partially overlap (Bovenschen et al. 2009). This was demonstrated by screening a library comprising 1000 fully randomised 15-amino acid peptides; no clear consensus sequence was found near the cleavage site, although both granzymes were shown to prefer P1Arg (Bovenschen et al. 2009). Cattle granzymes A, O and K and pig granzyme A are predicted to have similar primary substrate specificity (trypsin-like activity). However, comparison of extended specificity-determining residues at S2 and S3 of granzyme A, O and K in both cattle and pig (shown in Table 4) revealed different amino acids both between granzymes and between species orthologues, indicating potential differences in their extended substrate specificities. Although human and mouse granzyme B exhibit a high level of sequence similarity, including conserved primary substrate-determining residues, and both are aspases, they exhibit divergent substrate preferences. This is a consequence of differences in the residues that determine extended substrate specificity. Using a phage display substrate assay, human and mouse granzyme B were shown to have distinct substrate preferences at the key P4, P2 and P2′ substrate residues because of the presence of divergent residues at the corresponding granzyme binding sites (Kaiserman et al. 2006). The detection of divergent amino acids in the S2 and S3 extended specificity-determining residues in cattle granzyme B suggests that they may also have different substrate preferences than those of the human and mouse orthologues.

The bovine chymotrypsin-like locus contains a number genes closely related to granzymes, including genes encoding duodenases located adjacent to granzyme B, one of which (DDN1) has still been erroneously reported as granzyme B in the bovine genome and by other recent studies (Van Meulder et al. 2013; Ohta et al. 2014). However, comparison of their amino acid sequences revealed different primary specificity-determining residues in the S1 pocket predicted to result in enzymatic functions distinct from granzyme B (data not shown). Comparison with other chymotrypsin-like proteins indicates duodenase genes are likely to have dual specificity with trypsin-like as well as chymotrypsin-like activity (Zamolodchikova et al. 2003). DDN1 was originally isolated from duodenal mucosa (Antonov et al. 1992; Zamolodchikova et al. 1995). It is found in epithelial cells and the ducts of Brunner’s gland and is a potential activator of enteropeptidase, as indicated by its ability to hydrolyse recombinant bovine pro-enteropeptidase, which is an important enzyme in the digestive protease cascade (Zamolodchikova et al. 1997).

In conclusion, the work described in this paper has identified the complement of functional granzyme genes in cattle, developed PCR assays to examine their expression, and demonstrated that they are all expressed in activated antigen-specific CD8 T cells. Comparisons of their amino acid sequences with orthologues in other species have identified differences in residues that determine extended substrate specificities, suggesting that they differ in their protein substrate specificities. These findings provide the basis for further work to examine their role in killing of target cells by CD8 T cells.

## Electronic supplementary material

ESM 1 (DOCX 1137 kb)
