# Supplementary material for: Identification and annotation of bovine granzyme genes reveals a novel granzyme encoded within the trypsin-like locus
Source: Immunogenetics. 2018 Jun 8;70(9):585–97. doi: 10.1007/s00251-018-1062-6 (PMC6096847; doi:10.1007/s00251-018-1062-6)
Supplement: Supplementary file 1 — (DOCX 1137 kb) [file 251_2018_1062_MOESM1_ESM.docx]

Yang et al. Supplementary


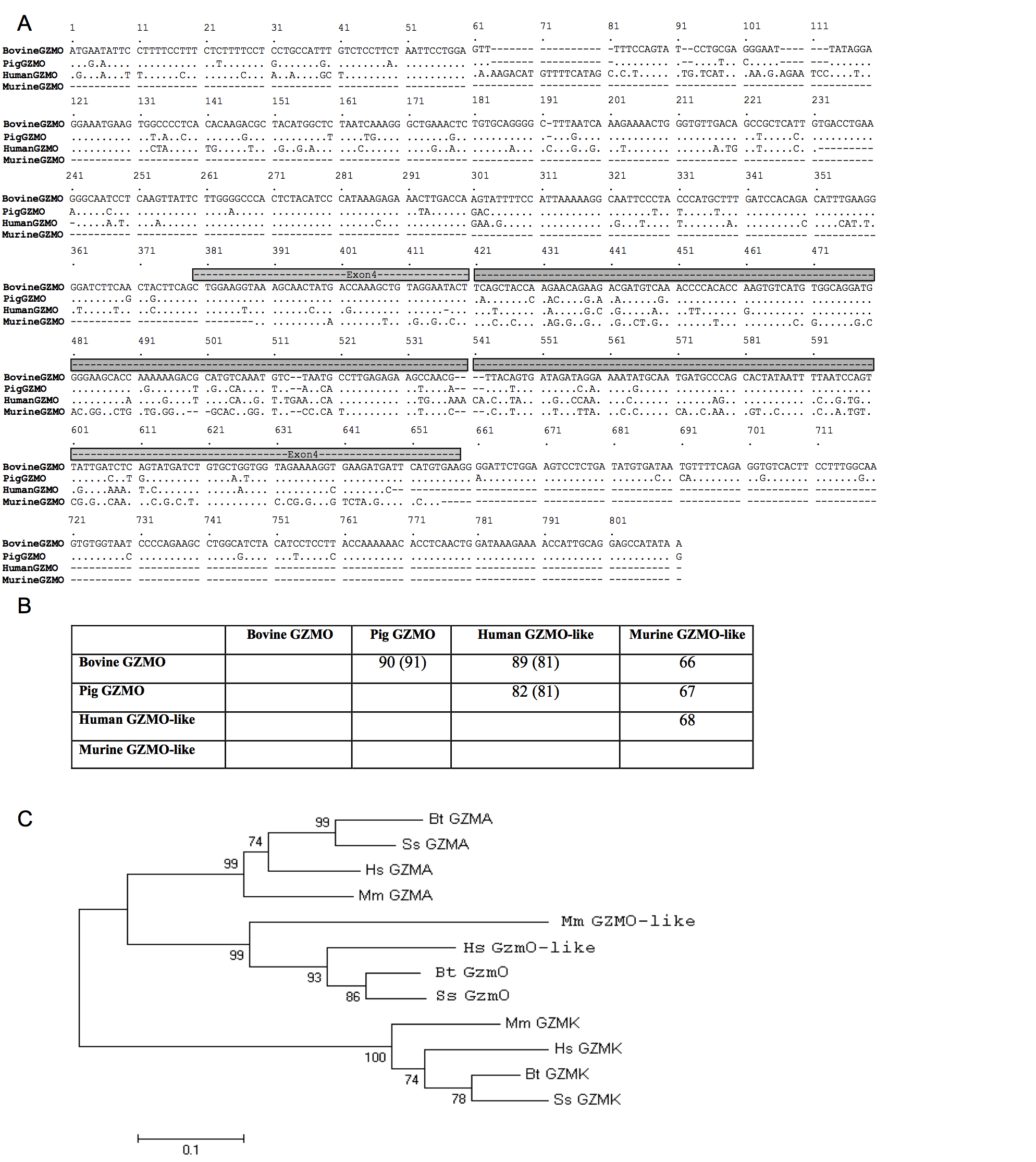


Supplementary 1. (A). Alignment of bovine, pig, human and murine granzyme O nucleotide sequences. Exon 4 is indicated by an asterisk. Dot-identical; Dash-gap. (B). Percentage nucleotide sequence similarities of granzyme O between species. The nucleotide similarity values are for exon 4 and those in brackets are for the available coding region sequences. (C). Phylogenetic relationships of human, mouse, cattle and pig trypsin-like granzyme genes. The nucleotide sequences were aligned by CLUSTAL W alignment with complete deletion of gaps for the analysis. The tree was constructed with the neighbour-joining algorithm using Mega 7.0 software. Numbers represent percentage bootstrap values out of 2,000 replications. The evolutionary distances were computed using the Maximum Composite Likelihood method and are in the units of the number of base substitutions per site. All positions containing gaps and missing data were eliminated. Hs - *Homo sapiens*; Mm - *Mus musculus*; Ss - *Sus scrofa*; Bt - *Bos Taurus*; GZM – granzyme.
